# Active control of dielectric nanoparticle optical resonance through electrical charging

**DOI:** 10.1038/s41598-022-13251-9

**Published:** 2022-06-16

**Authors:** Xuebang Gao, Li Xie, Jùn Zhou

**Affiliations:** 1 College of Civil Engineering and Mechanics, Lanzhou University, Lanzhou, 730000 China; 2 Key Laboratory of Mechanics on Disaster and Environment in Western China, Ministry of Education, Lanzhou University, Lanzhou, 730000 China

**Keywords:** Nanoscience and technology, Optics and photonics, Physics

## Abstract

A novel method for active control of resonance position of dielectric nanoparticles by increasing the excess charges carried by the nanoparticles is proposed in this paper. We show that as the excess charges carried by the particle increase, the oscillation frequency of excess charges will gradually increase, when it is equal to the incident frequency, resonance occurs due to resonant excitation of the excess charges. What is more, the formula of charges carried by an individual particle required to excite the resonance at any wavelength position is proposed. The resonance position can be directly controlled by means of particle charging, and the enhancement of resonance intensity is more obvious. This work has opened new avenues for the active control of plasmon resonances, which shows great promise for realizing tunable optical properties of dielectric nanoparticles.

## Introduction

Active control of the optical resonance of nanoparticles is of paramount importance to sensing, photocatalysis, photodetection and many other technological applications^1,2^. Active tuning of the resonance positions can be realized by altering the following parameters: the size and shape of particles^3–5^, the core-to-shell ratio^6,7^, the distance between nanoparticles^8^, material^9^, semiconductor and electronic doping^10–13^, surrounding dielectric material^14,15^, applying an electric field^16,17^, and charges of the nanoparticles^18^. Although the resonance positions of the nanoparticles can be actively tuned by changing the above-mentioned parameters, most of such methods have been irreversible so far ^1^. Therefore, significant challenges still remain for active control of the optical resonance of nanoparticles.

**Figure 1:**
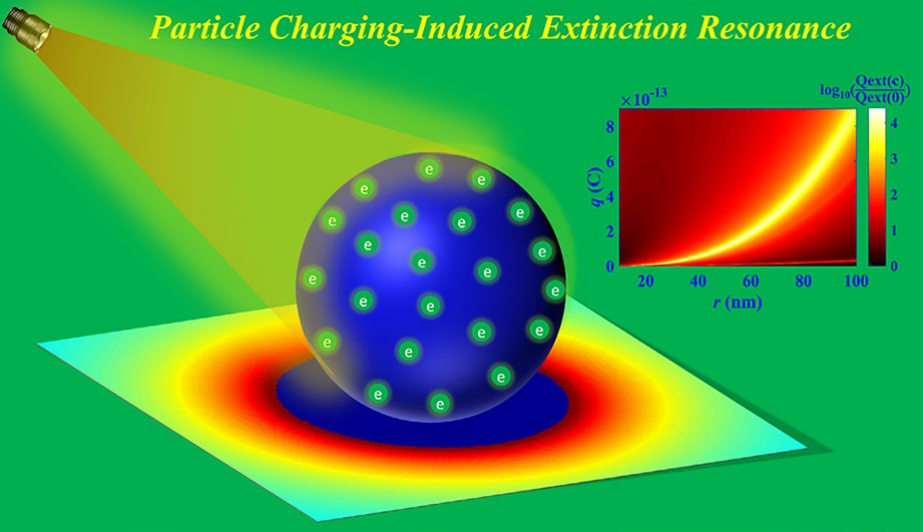
Charge-induced extinction resonance of dielectric nanoparticles.

Fortunately, charge-induced resonance shifts of extinction spectra due to changes in the free electron density of noble metals nanoparticles have been reported as a reversible process in recent years^3,19,20^. However, this optical resonance can only be blue-shifted by metal nanoparticles plasma charging^18^. But in many actual applications, nanoparticles are often designed for absorption and scattering in specific wavelength regions^21,22^, and it is currently still difficult to flexibly control the resonance position of metal nanoparticles through plasma charging. In addition, compared to the high cost and rarity of precious metals, the optical resonance of the dielectric nanoparticles caused by charges may have wide application prospects. Indeed, many dielectric particles are prone to being charged^23–25^, and the atmospheric pressure plasma jet can precisely control how many charges are carried by a particle^26^, and remain charged for up to 1 week^18^, making active control of dielectric nanoparticle optical resonance position by charging quite promising. Motivated by this, optical resonances by charged dielectric nanoparticles within the visible and near-infrared wavelength regions are investigated in this paper, as shown in Fig. 1, to find a novel method to actively control the resonance position of nanoparticles.

## Model description

**Figure 2:**
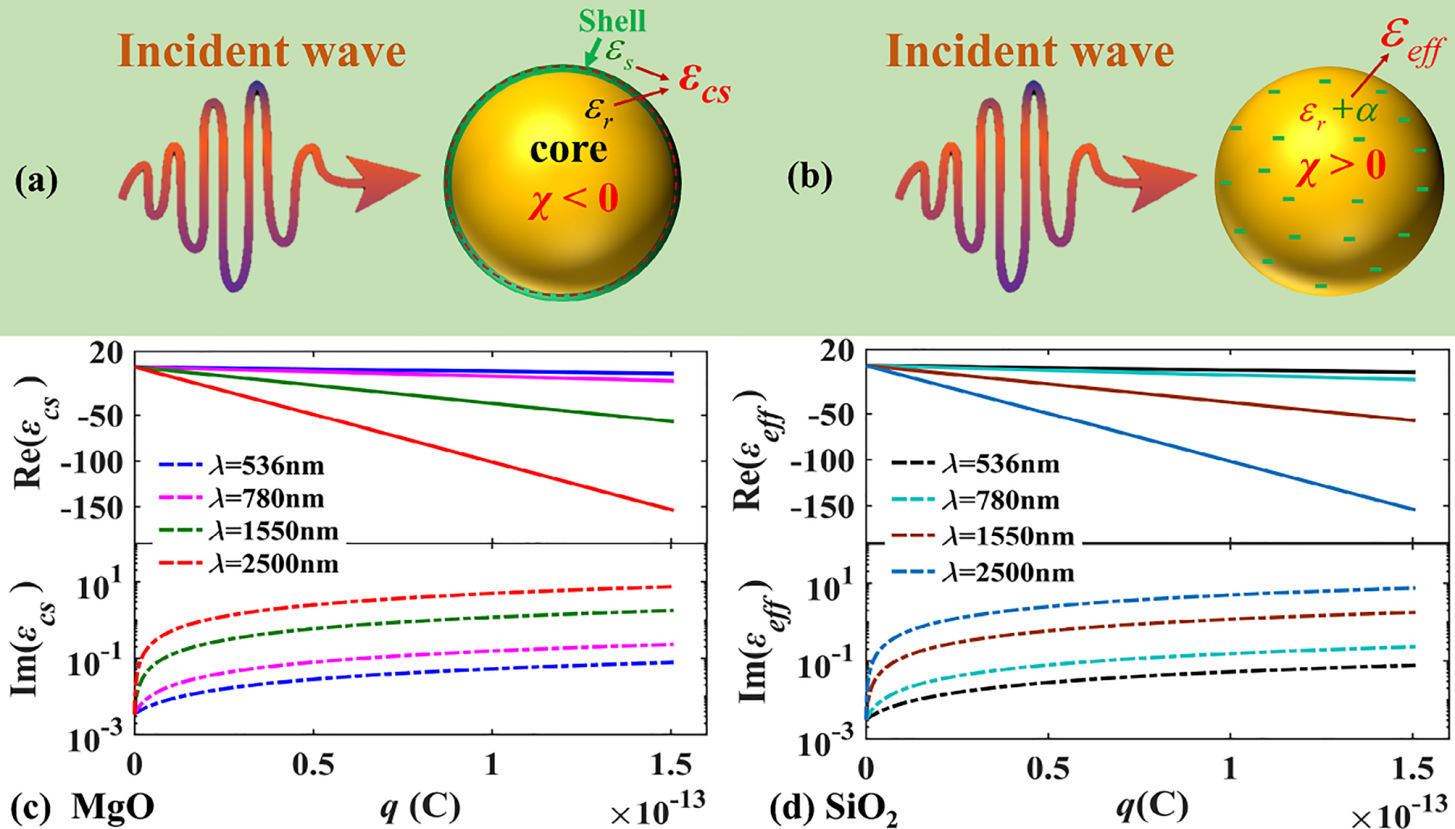
A schematic diagram of light irradiating on charged particles with (**a**) *χ* < 0 and (**b**) *χ* > 0, and real and imaginary parts of the charges-dependent function of (**c**) MgO and (**d**) SiO_2_, the radius of the charged particle *r* = 20 nm.

For any charged dielectric nanoparticle with radius *r* illuminated by light, its extinction depends on the electron affinity *χ* of the particle, because the existing form of the excess charges carried by the nanoparticle is different due to the different electron affinity *χ* ^27^, as shown in Fig. 2. For the particles of *χ* < 0 like MgO particles, the excess charges carried by the particles are limited to a shell of the atomic scale on the surface of the particles^28^, shown as in Fig. 2a, while for the micro-nano sized particles of *χ* > 0, like SiO_2_ particles, the excess electrons will be uniformly distributed in the particles^29^, shown as in Fig. 2b. The optical properties of charged dielectric nanoparticles can be studied by the core–shell model for particles of *χ* < 0 and by the equivalent model for particles of *χ* > 0.

For the particles of *χ* < 0, the charges will be limited within shell with a thickness of *t*_*s*_ ($t_{s} = 2{\text{\AA}}$^28^), so the radius of the particle core $r_{1} = r - t_{s}$. When the light or near-infrared wave irradiates on a spherical particle with such a core–shell structure, the corresponding size parameters of the whole particle and the core are $y = {2}\pi r{/}\lambda$ and $x = {2}\pi r_{1} {/}\lambda$, respectively, in which *λ* is the wavelength and the corresponding angular frequency *ω* = 2*πc*/*λ*, and *c* is light speed in vacuum, 3.0 × 10^8^ m/s. The permittivity of the shell can be reorganized as

1 $$\varepsilon_{s} { = }\varepsilon_{r} { + }i\sigma_{s} /\omega \varepsilon_{0}$$

and the dielectric constant of the corresponding core $\varepsilon_{r} = \varepsilon_{real} + i\varepsilon_{imag}$. $\sigma_{s}$ is the bulk conductivity of the shell, $\sigma_{s} { = }ie^{2} n_{s} /m_{e} (\omega + i\gamma )$, in which $i=\sqrt{-1}$, and $n_{s}$ is the electron density of the shell, $n_{s} = 3q/\left[ {4\pi (r^{3} - r_{1}^{3} )e} \right]$, where *q* is the net charge of the particle; *e* is the charge of a single electron, *e* = 1.6 × 10^–19^ C; $m_{e}$ is the mass of the electron, $m_{e} = 9.109 \times 10^{ - 31} {\text{kg}}$; $\gamma$ is the damping coefficient^30^, and $\gamma { = }\gamma_{c} k_{B} T/\hbar$, with the Boltzmann constant $k_{B} = {1}.{38} \times {1}0^{{ - {23}}} {\text{JK}}^{{ - {1}}}$ and the reduced Planck constant $\hbar { = 1}.0{546} \times {1}0^{{ - {34}}} {\text{Js}}$ and the temperature *T*. Further the permittivity of the charge shell can be reorganized as

2 $$\varepsilon_{s} { = }\varepsilon_{r} - \omega_{s}^{2} /(\omega^{2} + i\omega \gamma )$$

where $\omega_{s} = \sqrt {n_{s} e^{2} /(m_{e} \varepsilon_{0} )}$ is the plasma frequency associated with collective oscillation of the excess charges, and *ε*_0_ = 8.85 × 10^–12^ F/m is the permittivity of vacuum. With the Drude model of dielectric function^31^, the oscillation frequency of excess charges carried by particles can be expressed as

3 $$\omega_{rs} = \omega_{s} /\sqrt { - {\text{Re(}}\varepsilon_{rs} ) + \varepsilon_{real} } \, \;{ (}\gamma \ll \omega {)}$$

in which ${\text{Re}} (\varepsilon_{rs} )$ is the real part of the permittivity of the shell $\varepsilon_{rs}$ at resonance. According to Ref. ^32^, the equivalent dielectric function of such charged particle is

4 $$\varepsilon_{CS} = \frac{{v_{C} }}{{v_{C} + v_{s} }}\varepsilon_{r} + \frac{{v_{s} }}{{v_{C} + v_{s} }}\varepsilon_{s}$$

in which ${v}_{c}$ is the volume of the core and ${v}_{s}$ is the volume of the shell.

According to Mie scattering theory^33^, the extinction efficiency of core–shell structure is given by

5 $$Q_{ext} {(}c{) = }\frac{2}{{y^{2} }}\sum\limits_{n = 1}^{\infty } {\left( {2n + 1} \right){\text{Re}} \left( {a_{n}^{r} + b_{n}^{r} } \right)}$$

where $a_{n}^{r} \,$ and $\, b_{n}^{r}$ are the Mie’s scattering coefficients (see “Supplementary material” for details).

For the charged particles of *χ* > 0, the excess electrons will be uniformly distributed in the particles^27^. Therefore, the effective dielectric function of this charged particle can be calculated by the dielectric constant *ε*_*r*_ of particle material and the polarizability of excess charges *α*

6 $$\varepsilon_{eff} = \varepsilon_{r} { + }\alpha = (\varepsilon_{real} + \alpha_{real} ) + i(\varepsilon_{imag} + \alpha_{imag} )$$

where $\alpha = i\sigma_{b} /\varepsilon_{0} \omega$ and *σ*_*b*_ is the electric conductivity contributed by the excess charges and it can be calculated by

7 $$\sigma_{b} = ie^{2} n_{b} /m_{e} (\omega + i\gamma )$$

in which $n_{b}$ denotes the number density of excessive electrons of the particle, $n_{b} = 3q/4\pi er^{3}$. Therefore, the polarizability can be organized as

8 $$\alpha { = } - \omega_{b}^{2} /(\omega^{2} + i\omega \gamma ) = - \frac{{\omega_{b}^{2} }}{{\omega^{2} + \gamma^{2} }} + i\frac{{\omega_{b}^{2} \gamma }}{{\omega^{3} + \omega \gamma^{2} }}$$

where $\omega_{b} = \sqrt {n_{b} e^{2} /m_{e} \varepsilon_{0} }$ is the surface plasma frequency. The oscillation frequency of excess charges carried by the particles is $\omega_{rb} = \omega_{b} {/}\sqrt {\varepsilon_{real} + 2}$ when *γ* ≪ *ω*. The refractive index $m = \sqrt {\varepsilon_{eff} }$ can be obtained through its effective dielectric function. Based on Mie scattering theory, the extinction efficiency of charged particle is given by

9 $$Q_{ext}^{{}} (c) = (2/x^{2} )\sum\limits_{n = 1}^{\infty } {(2n + 1){\text{Re}} (a_{n} + b_{n} )}$$

where *a*_*n*_ and *b*_*n*_ are scattering coefficients obtained by Bohren and Huffman ^33^.

## Results and discussions

For nano-scale particles, as the charges carried by the particle increasing, the material properties are changed as well, and some of their properties are not the same as ones of the corresponding neutral particle anymore, such as the equivalent dielectric function of individual charged SiO_2_ and MgO particles shown as in Fig. 2c,d. In Fig. 2c,d, four representative wavelengths in the visible (*λ* = 536 nm and 780 nm) and infrared bands (1550 nm and 2500 nm) have been selected, and the radius of SiO_2_ and MgO particles are 20 nm. From Fig. 2c,d, it can be found that with the increase of the charges carried by the particles, the imaginary part of the equivalent dielectric function increases gradually, while the real part of the equivalent dielectric function gradually decreases. The reason is that the increase of excess charges results in an increase in the density of excess electrons on the particle, and then an increase in excess electrons density further affects the equivalent dielectric function of charged nanoparticles, which was also demonstrated by Juluri et al.^3^. In addition, the longer the wavelength is, the greater the effect of the excess charges on the equivalent dielectric function is. With *λ* = 2500 nm, the real part of the particle's equivalent dielectric function can even reach − 154 to see Fig. 2c,d.

**Figure 3:**
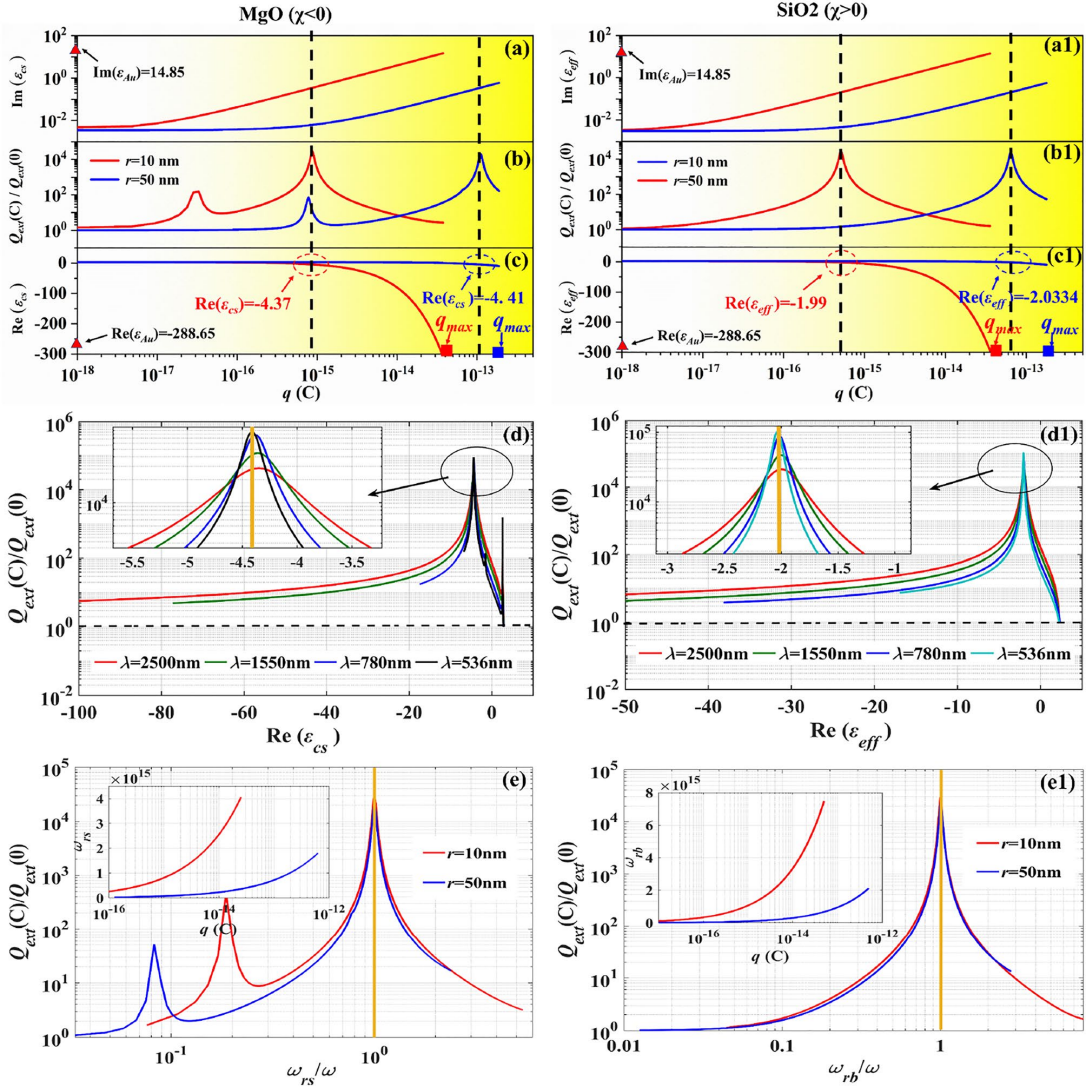
For MgO and SiO_2_ particles with radius of *r* = 10 nm and *r* = 50 nm, with the increase of the charges carried by the particles, the imaginary part (**a**,**a1**) and the real part (**c**,**c1**) of the equivalent dielectric function of MgO and SiO_2_ particles are changed, in which the wavelength is 2500 nm. The variation of $Q_{ext} (c)/Q_{ext} (0)$ with the quantity of charge carried by particles (**b**,**b1**) and the ratio of resonance frequency of excess charges to incident frequency (**e**,**e1**). $Q_{ext} (c)/Q_{ext} (0)$ of (**d**) MgO particle and (**d1**) SiO_2_ particle varying with the real part of the equivalent dielectric in case of *λ* = 536 nm, 780 nm, 1550 nm and 2500 nm, and *r* = 10 nm.

Next, for the given incident wave illustrating on a charged particle, the variation of *Q*_*ext*_(*c*)/*Q*_*ext*_(0) (the ratio of the extinction by a charged particle to the one by the corresponding neutral particle), as well as the corresponding imaginary and real parts of the equivalent dielectric function with the charges carried by the particle are shown in Fig. 3a–c (MgO particle) and Fig. 3a1–c1 (SiO_2_ particle). From Fig. 3b,b1, it can be found that the extinction resonance can occur during the particle charging process for both MgO particle and SiO_2_ particle although these two particles are with different electron affinity, in which the incident wavelength is 2500 nm. Compare Fig. 3b with Fig. 3c, for MgO particles with *r* = 10 nm and *r* = 50 nm, and it can be seen that the extinction resonance occurs at *q* = 8.72 × 10^–16^ C and *q* = 1.11 × 10^–13^ C, respectively, and in such situation, it’s obvious to find that the real part of the equivalent permittivity of both particles Re(*ε*_*cs*_) ≈ − 4.3; again compare Fig. 3b1 with Fig. 3c1, for SiO_2_ particles of *r* = 10 nm and *r* = 50 nm, extinction resonance occurs at *q* = 5.1 × 10^–16^ C and 6.43 × 10^–14^ C, with the corresponding real part of the equivalent permittivity of both these particles Re (*ε*_*eff*_) ≈-2. That means the real part of the equivalent dielectric function can be used to determine if the extinction resonance can be occurred for a given particle when the particle carried excess charges, which also is verified in case of *λ* = 536 nm, 780 nm, 1550 nm and 2500 nm shown as in Fig. 3d,d1 (*r* = 10 nm).

Further, let’s reveal why the extinction resonance occurs during the particle charging process. As excess charges density increases, the oscillation frequency of the excess charge has been changed, as shown in the insert in Fig. 3e,e1. It can be found that the oscillation frequency increases with the increase of excess charges carried by the particle, and extinction resonance occurs if only the oscillation frequencies of the excess charges are equal to the incident wave frequencies to see the Fig. 3e,e1. This reveals that for a charged dielectric nanoparticle, whether with positive electron affinity or negative electron affinity, the extinction resonance can occur, because when the oscillation frequency of the excess charges carried by the particle close to the frequency of the incident electromagnetic wave, it will result in resonant excitation. In addition, due to the quadrupole resonance, a second resonance is also observed in Fig. 3b at excess charges between 10^–16^ C and 10^–17^ C, which is related to the thickness of the shell.

**Figure 4:**
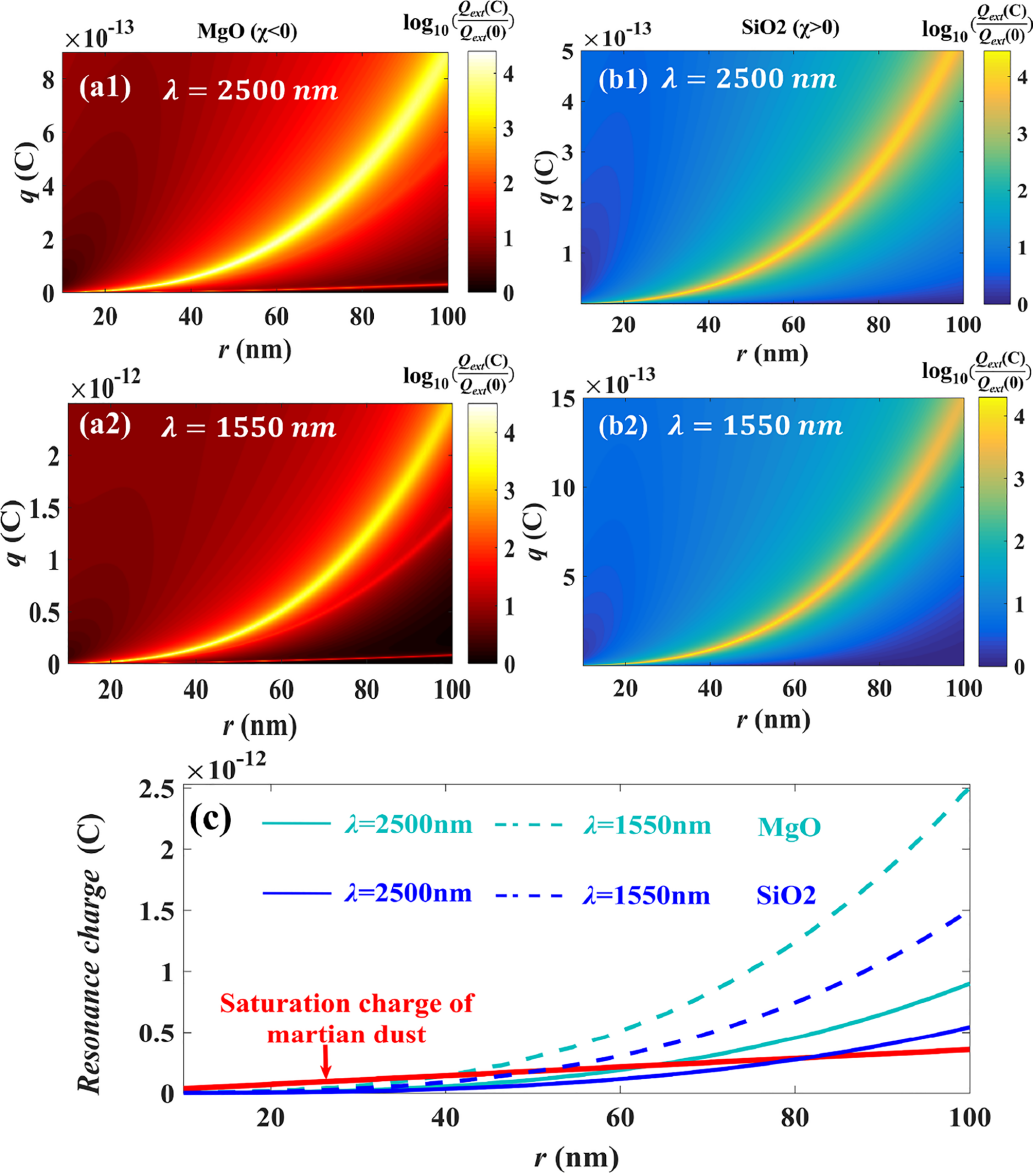
The contour of $\log_{10} (Q_{ext} (c)/Q_{ext} (0))$ varying with the particle size and the excess charges, in which wavelengths are *λ* = 2500 nm and *λ* = 1550 nm, (**a1**,**a2**) MgO particle, (**b1**,**b2**) SiO_2_ particle. (**c**) The quantity of charge required for resonance to occur with varying particle sizes at different wavelengths, and the red solid lines mean the saturation charges of particles with given particle size.

Further the contour maps of $\log_{10} (Q_{ext} (c)/Q_{ext} (0))$ of the charged MgO particle and the SiO_2_ particle are displayed with particle size and excess charges at two wavelengths (*λ* = 2500 nm and *λ* = 1550 nm) shown as in Fig. 4a1–b2. It can be found that given the wavelength, for different particle sizes of dielectric nanoparticles, the extinction resonance can be occurred if only that the particle can carry proper charges shown as the bright band in Fig. 4a1–b2. Further, for given particle size and selected resonance position, the quantity of charges carried by individual particle to excite extinction resonance is derived as,

10 $${q}_{resonance}=\frac{4\pi {\varepsilon }_{0}{m}_{e}({\varepsilon }_{real}-{Re(\varepsilon }_{rs}))}{3e}{({r}^{3}-{r}_{1}^{3})\omega }^{2} (\chi <0)$$

11 $${q}_{resonance}=\frac{4\pi {\varepsilon }_{0}{m}_{e}\left({\varepsilon }_{real}+2\right)}{3e}{{r}^{3}\omega }^{2} (\chi >0)$$

For the selected resonance position, *q*_*resonance*_ to excite the resonance directly depends on the particle size shown as in Fig. 4c. While for given the particle size, the charges to excite resonance at *λ* = 1550 nm is more than the ones at *λ* = 2500 nm. Theoretically, for any selected resonance position, if the particle size is given, the charges to excite the resonance can be calculated by formula (10) or (11). However, the quantity of charges carried by an individual particle is limited by breakdown voltage which is related to the particle material, ambient pressure, etc.^34^. Actually the charges carried by an individual particle cannot be infinite and have a saturation value, e.g. the saturation charges of Martian dust shown as the red line in Fig. 4c ^35^. Therefore, if the charges required to excite the resonance are beyond the saturation value, the resonance cannot be occurred, while if the charges less than the corresponding saturation value the resonance can be realized such as MgO particles smaller than 63 nm, SiO_2_ particles smaller than 82 nm, the extinction resonance can be occurred when *λ* = 2500 nm. In addition, it can be found from Fig. 4c that the resonance charge required to excite the resonance is less for SiO_2_ particle than for MgO particle. Therefore, the resonant charge formula given here can be referred to in practical applications to select a more suitable particle material to achieve resonance at less resonant charge.

**Figure 5:**
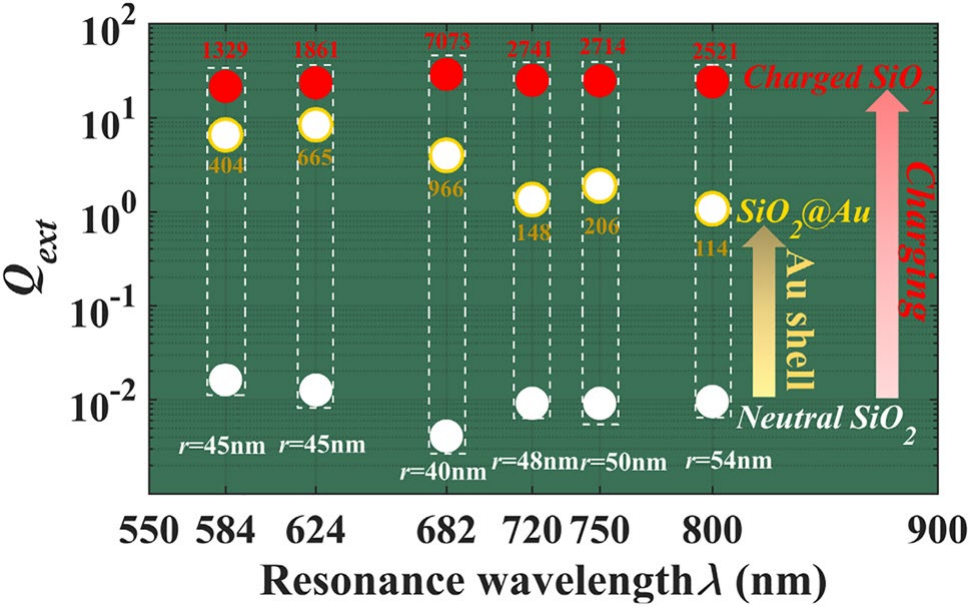
The extinction efficiency of neutral SiO_2_ nanoparticles (white spheres), SiO_2_@Au (SiO_2_ nanoparticles wrapping Au shell) core–shell nanoparticles (white spheres core yellow shell), charged SiO_2_ nanoparticles (red spheres) at the resonance positions, the numbers above the red sphere and below the core–shell sphere indicate the magnification of extinction amplification by charges and by Au shell, respectively.

Finally, the extinction efficiency of the charged SiO_2_ nanoparticles at resonance position are calculated as shown in Fig. 5, which is also compared with the extinction resonance values of SiO_2_@Au nanoparticles. In Fig. 5, the white circles indicate the extinction efficiency of neutral SiO_2_ particles with given sizes, and the white core with yellow shell indicate the extinction efficiency of SiO_2_@Au nanoparticles at the resonance position, and at the same resonance position, the red spheres indicate the extinction efficiency of charged SiO_2_ particles. Here, parameters such as resonance position, SiO_2_ core radius and Au shell thickness are selected from existed studies^6,9,21,36,37^, as detailed in Tables S1 and S2 in the “Supplementary material”. From Fig. 5, it can be found the charge-induced resonance is much higher than the one by coating Au of a particle with same size at same resonance wavelength. Actually, the maximum extinction efficiencies of pure Au nanoparticles and charged SiO_2_ nanoparticles are also compared at the same particle size and resonance position, and the former is much lower than the latter, to see Table S1 in “Supplementary material”. That means the charge-induced resonance can not only precisely control the resonance position, but also improve the intensity of extinction resonance.

## Conclusions

To conclude, a new method to actively control optical resonance by increasing the quantity of charges carried by the dielectric nanoparticles is proposed in this paper. Our results suggest that extinction resonance can be generated by increasing the particle charges in the visible and infrared bands, and infer that it is caused by the resonant excitation of excess charges carried by the particle. Furthermore, the quantity of charge required for exciting resonance is given, which is a function of the nanoparticle size, wavelength, and dielectric properties of particles. Finally, dielectric nanoparticles can resonate at any wavelength position by particle charging and the generation of larger extinction resonance values. Therefore, this method can be used to realize a flexibly adjustable optical resonance, and may be promising for controlling the optical resonance of nanoparticles in technical applications, without including the high-cost or noble metal.

## Supplementary Information

Supplementary Information.

## Data Availability

The data that support the findings of this study are available from the corresponding author upon reasonable request.
