# Supplementary material for: Active control of dielectric nanoparticle optical resonance through electrical charging
Source: Sci Rep. 2022 Jun 16;12:10117. doi: 10.1038/s41598-022-13251-9 (PMC9203548; doi:10.1038/s41598-022-13251-9)
Supplement: Supplementary file 1 — Supplementary Information. [file 41598_2022_13251_MOESM1_ESM.pdf]

Supplementary Materials for

Active control of dielectric nanoparticle optical resonance through electrical charging

Xuebang Gao, Li Xie and Jùn Zhou

[1]College of Civil Engineering and Mechanics, Lanzhou University, Lanzhou 730000, China;

[2]Key Laboratory of Mechanics on Disaster and Environment in Western China, Ministry of Education, Lanzhou 730000, China.

This PDF file includes:

1. For dielectric core charged shell spheres, the derivation of Mie scattering coefficients.

When the plane harmonic EMW is incident a charged particle described by the core-shell model shown as in FIG.1, the wave equations are

$$\left.\begin{aligned}\nabla^2\mathbf{E}_1+k^2\mathbf{E}_1=0\\ \nabla^2\mathbf{H}_1+k^2\mathbf{H}_1=0\end{aligned}\right\} \text{ and } \left.\begin{aligned}\nabla^2\mathbf{E}_2+k^2\mathbf{E}_2=0\\ \nabla^2\mathbf{H}_2+k^2\mathbf{H}_2=0\end{aligned}\right\} \qquad (1)$$

in which the subscriptions 1 and 2 study for the filed component in particle bulk and in shell layer. The fields $\mathbf{E}_1$, $\mathbf{H}_1$ in particle material and the fields $\mathbf{E}_2$, $\mathbf{H}_2$ can be expanded by spherical harmonic vector serials $\mathbf{M}_{o1n}$, $\mathbf{N}_{o1n}$, $\mathbf{M}_{e1n}$, $\mathbf{N}_{e1n}$,

$$\left.\begin{aligned}\mathbf{E}_1&=\sum_{n=1}^{\infty}E_n(c_n\mathbf{M}_{o1n}^{(1)}-id_n\mathbf{N}_{e1n}^{(1)})\\ \mathbf{H}_1&=\frac{-k_1}{\omega\mu_1}\sum_{n=1}^{\infty}E_n(d_n\mathbf{M}_{o1n}^{(1)}+ic_n\mathbf{N}_{e1n}^{(1)})\end{aligned}\right\} \qquad (2)$$

$$\left.\begin{aligned}\mathbf{E}_2&=\sum_{n=1}^{\infty}E_n[f_n\mathbf{M}_{o1n}^{(1)}-ig_n\mathbf{N}_{e1n}^{(1)}+v_n\mathbf{M}_{o1n}^{(2)}\text{-i}w_n\mathbf{N}_{o1n}^{(2)}]\\ \mathbf{H}_2&=-\frac{k_2}{\omega\mu_2}\sum_{n=1}^{\infty}E_n[g_n\mathbf{M}_{e1n}^{(1)}+if_n\mathbf{N}_{o1n}^{(1)}+w_n\mathbf{M}_{e1n}^{(2)}+\text{i}v_n\mathbf{N}_{o1n}^{(2)}]\end{aligned}\right\} \qquad (3)$$

in which $E_n=|\mathbf{E}_i|i^n(2n+1)/n(n+1)$, μ,μ_1,μ_2 are the permeabilities of the surrounding medium, core, and shell.

And the scattering fields $\mathbf{E}_s$, $\mathbf{H}_s$ also can be expanded by spherical harmonic vector serials,

$$\left.\begin{aligned}\mathbf{E}_s &= \sum_{n=1}^{\infty} E_n (ia_n \mathbf{N}_{e1n}^{(3)} - b_n \mathbf{M}_{o1n}^{(3)}) \\ \mathbf{H}_s &= \frac{k}{\omega\mu} \sum_{n=1}^{\infty} E_n (ib_n \mathbf{N}_{o1n}^{(3)} + a_n \mathbf{M}_{e1n}^{(3)})\end{aligned}\right\} \qquad (4)$$

The superscripts (1), (2) and (3) of spherical harmonic vector in (11)-(13) study for the first kind/second/third spherical Bessel functions, separately. On the boundary between the particle material and the Shell, the boundary conditions are

$$\left.\begin{aligned}(\mathbf{E}_2 - \mathbf{E}_1) \times \vec{n} = 0 \\ (\mathbf{H}_2 - \mathbf{H}_1) \times \vec{n} = 0\end{aligned}\right\} \qquad (5)$$

On the boundary between shell and surrounding environment, boundary conditions are

$$\left.\begin{aligned}(\mathbf{E}_i + \mathbf{E}_s - \mathbf{E}_2) \times \vec{n} = 0 \\ (\mathbf{H}_i + \mathbf{H}_s - \mathbf{H}_2) \times \vec{n} = 0\end{aligned}\right\} \qquad (6)$$

The scattering field can be solved by Mie scattering theory and Mie's scattering coefficients, a_n and b_n are as following

$$\begin{aligned}a_n^r &= \frac{\psi_n(y)\left[\psi_n'(m_2 y) - A_n \chi_n'(m_2 y)\right] - m_2 \psi_n'(y)\left[\psi_n(m_2 y) - A_n \chi_n(m_2 y)\right]}{\xi_n(y)\left[\psi_n'(m_2 y) - A_n \chi_n'(m_2 y)\right] - m_2 \xi_n'(y)\left[\psi_n(m_2 y) - A_n \chi_n(m_2 y)\right]} \\ b_n^r &= \frac{m_2 \psi_n(y)\left[\psi_n'(m_2 y) - B_n \chi_n'(m_2 y)\right] - \psi_n'(y)\left[\psi_n(m_2 y) - B_n \chi_n(m_2 y)\right]}{m_2 \xi_n(y)\left[\psi_n'(m_2 y) - B_n \chi_n'(m_2 y)\right] - \xi_n'(y)\left[\psi_n(m_2 y) - B_n \chi_n(m_2 y)\right]}\end{aligned} \qquad (7)$$

in which A_n and B_n are of the following form,

$$\begin{aligned}A_n &= \frac{m_2 \psi_n(m_2 x)\psi_n'(m_1 x) - m_1 \psi_n'(m_2 x)\psi_n(m_1 x)}{m_2 \chi_n(m_2 x)\psi_n'(m_1 x) - m_1 \chi_n'(m_2 x)\psi_n(m_1 x)} \\ B_n &= \frac{m_2 \psi_n(m_1 x)\psi_n'(m_2 x) - m_1 \psi_n(m_2 x)\psi_n'(m_1 x)}{m_2 \chi_n'(m_2 x)\psi_n(m_1 x) - m_1 \chi_n(m_2 x)\psi_n'(m_1 x)}\end{aligned} \qquad (8)$$

where $y = 2\pi R/\lambda$. ψ_n, ξ_n and χ_n are Riccati-Bessel functions. The refractive indices of the bare sphere and the conducting layer are denoted by $m_1 = \sqrt{\varepsilon_r}$ and m_2, respectively. For the charged layer, $m_2 = \sqrt{\varepsilon_2}$, where $\varepsilon_2 = \varepsilon_r + i\frac{\sigma_{cond}}{\omega\varepsilon_0}$ is the dielectric constant of the charged layer.

Table S1. Dielectric constants of **MgO** and **SiO2** particles[1-3]

**Wavelength**	**Dielectric constant MgO**	**Dielectric constant SiO2**
λ=536 nm	3.034+0.0i	2.151+0.0057i
λ=584 nm	3.02+0.0i	2.146+0.0053i
λ=624 nm	3.011+0.0i	2.142+0.0049i
λ=682 nm	3.0+0.0i	2.139+0.0045i
λ=720 nm	2.994+0.0i	2.137+0.0042i
λ=750 nm	2.99+0.0i	2.136+0.004i
λ=780 nm	2.987+0.0i	2.135+0.0039i
λ=800 nm	2.985+0.0i	2.134+0.0038i
λ=1550 nm	2.94+0.0i	2.124+0.002i
λ=2500 nm	2.89+0.0i	2.146+0.00i

Table S2. For SiO_2@Au core-shell nanoparticles, the resonance wavelength, the core radius and the shell layer thickness were selected from different literature.

Resonance wavelength	***SiO_2 core radius* (nm)**	***Au shell thickness* (nm)**	**References**
λ=584 nm	45	20	Saini et al [4]
λ=624 nm	45	13	Saini et al [4]
λ=682 nm	40	12	Bardhan et al [5]
λ=720 nm	48	22	Hirsch et al [6]
λ=750 nm	50	10	Lu et al [7]
λ=800 nm	54	12	Gobin et al [8]

Table S3. Maximum extinction efficiency of Au nanoparticles and charged SiO_2 nanoparticles at different wavelengths and particle sizes.

Frequency	Au nanospheres[9]		Charged SiO_2	
	radii	$Q_{maximum}$	radii	$Q_{maximum}$
**λ=521 nm**	10 nm	0.98	10 nm	30
**λ=528 nm**	20 nm	2.47	20 nm	40.43
**λ=549 nm**	40 nm	6	40 nm	23.73

References

[1] R. E. Stephens and I. H. Malitson, Journal of Research of the National Bureau of Standards **49**, 249 (1952).

[2] L. Gao, F. Lemarchand, and M. Lequime, Optics express **20**, 15734 (2012).

[3] L. V. Rodríguez-de Marcos, J. I. Larruquert, J. A. Méndez, and J. A. Aznárez, Optical Materials Express **6**, 3622 (2016).

[4] A. Saini, T. Maurer, I. I. Lorenzo, A. R. Santos, J. Béal, J. Goffard, D. Gérard, A. Vial, and J. Plain, Plasmonics **10**, 791 (2015).

[5] R. Bardhan, N. K. Grady, T. Ali, and N. J. Halas, ACS nano **4**, 6169 (2010).

[6] L. R. Hirsch, J. B. Jackson, A. Lee, N. J. Halas, and J. L. West, Analytical Chemistry **75**, 2377 (2003).

[7] Y. Lu, J. Zhong, G. Yao, and Q. Huang, Sensors and Actuators B: Chemical **258**, 365 (2018).

[8] A. M. Gobin, M. H. Lee, N. J. Halas, W. D. James, R. A. Drezek, and J. L. West, Nano Letters **7**, 1929 (2007).

[9] P. K. Jain, K. S. Lee, I. H. El-Sayed, and M. A. El-Sayed, The Journal of Physical Chemistry B **110**, 7238 (2006).
